# Modification of Cyclic NGR Tumor Neovasculature-Homing Motif Sequence to Human Plasminogen Kringle 5 Improves Inhibition of Tumor Growth

**DOI:** 10.1371/journal.pone.0037132

**Published:** 2012-05-10

**Authors:** Weiwei Jiang, Guanghui Jin, Dingyuan Ma, Feng Wang, Tong Fu, Xiao Chen, Xiwen Chen, Kunzhi Jia, Faiz M. M. T. Marikar, Zichun Hua

**Affiliations:** 1 The State Key Laboratory of Pharmaceutical Biotechnology and School of Stomatology, Affiliated Stomatological Hospital, Nanjing University, Nanjing, People's Republic of China; 2 Department of Basic Medical Sciences, Medical College, Xiamen University, Xiamen, People's Republic of China; 3 Department of Nuclear Medicine, The Affiliated Nanjing First Hospital, Nanjing Medical University, Nanjing, People's Republic of China; 4 Changzhou High-Tech Research Institute of Nanjing University, Changzhou, People's Republic of China; University of Chicago, United States of America

## Abstract

**Background:**

Blood vessels in tumors express higher level of aminopeptidase N (APN) than normal tissues. Evidence suggests that the CNGRC motif is an APN ligand which targets tumor vasculature. Increased expression of APN in tumor vascular endothelium, therefore, offers an opportunity for targeted delivery of NGR peptide-linked drugs to tumors.

**Methods/Principal Findings:**

To determine whether an additional cyclic CNGRC sequence could improve endothelial cell homing and antitumor effect, human plasminogen kringle 5 (hPK5) was modified genetically to introduce a CNGRC motif (NGR-hPK5) and was subsequently expressed in yeast. The biological activity of NGR-hPK5 was assessed and compared with that of wild-type hPK5, *in vitro* and *in vivo*. NGR-hPK5 showed more potent antiangiogenic activity than wild-type hPK5: the former had a stronger inhibitory effect on proliferation, migration and cord formation of vascular endothelial cells, and produced a stronger antiangiogenic response in the CAM assay. To evaluate the tumor-targeting ability, both wild-type hPK5 and NGR-hPK5 were ^99 m^Tc-labeled, for tracking biodistribution in the *in vivo* tumor model. By planar imaging and biodistribution analyses of major organs, NGR-hPK5 was found localized to tumor tissues at a higher level than wild-type hPK5 (approximately 3-fold). Finally, the effects of wild-type hPK5 and NGR-modified hPK5 on tumor growth were investigated in two tumor model systems. NGR modification improved tumor localization and, as a consequence, effectively inhibited the growth of mouse Lewis lung carcinoma (LLC) and human colorectal adenocarcinoma (Colo 205) cells in tumor-bearing mice.

**Conclusions/Significance:**

These studies indicated that the addition of an APN targeting peptide NGR sequence could improve the ability of hPK5 to inhibit angiogenesis and tumor growth.

## Introduction

*In vivo* panning of phage libraries in tumor-bearing animals has proved useful for selecting peptides able to interact with proteins expressed within tumor-associated vessels and to home to neoplastic tissues [1]. Among the targeting probes identified thus far, a peptide containing the NGR motif is an aminopeptidase N (CD13) ligand that targets tumor vasculature [2].

Numerous studies have focused on the use of the NGR motif for ligand-directed delivery of various drugs and particles to tumor vessels [3], such as tumor necrosis factor α (TNF α) [4], doxorubicin [5], proapoptotic peptides [6], liposome [7] and tissue factor [8]–[10]. For example, the antitumor activity of NGR-TNF α in animal models was 10–30 times stronger than that of wild-type TNF α, whereas their toxicities were similar [4]. It has also been reported that NGR modification of antiangiogenic molecules, such as endostatin, could improve tumor localization and, in consequence, effectively inhibited ovarian carcinoma growth in athymic nude mice [11], indicating that addition of a vascular targeting sequence NGR could enhance the biological activity of an antitumor or antiangiogenic molecule.

Antiangiogenic therapy for solid tumors clearly destroys tumor vasculature and reduces tumor growth [12]. Extensive research has led to the identification and isolation of several regulators of angiogenesis, some of which represent therapeutic targets [12], [13]. Human plasminogen kringle 5 (hPK5), a proteolytic fragment of plasminogen, is an endogenous angiogenic inhibitor [12]–[14]. Recombinant hPK5 displays the most potent inhibitory activity to endothelial cell proliferation and migration [14]–[16] among naturally occurring angiogenesis inhibitors. A recombinant hPK5 has also been shown to induce apoptosis in proliferating endothelial cells and tumor cells [17], [18]. Because of its high efficacy, cell type selectivity, and small molecular weight, hPK5 has considerable potential in the treatment of neovascular diseases involving solid tumors [12], [13], [19]. A number of earlier studies have suggested that tumor suppression by hPK5 depends on its antiangiogenic activity and hPK5 could have therapeutic potential in hepatocellular carcinoma [20]–[22], lung cancer [23], [24], glioblastoma [25], [26] and ovarian cancer [27]. Moreover, several reports including our previous investigation have also indicated that combination of hPK5 with other therapeutic agents, such as ionizing radiation [26], [28] and matrix metalloproteinase [29] could remarkably enhance the antiangiogenic effect during tumor formation. These findings prompted us to deliver hPK5 to the tumor by a vascular-targeting approach.

To determine whether an additional NGR sequence could improve endothelial cell homing and biological activity, hPK5 was modified genetically to introduce an NGR motif and was expressed in the yeast host strain GS115. Our studies showed that NGR-hPK5 was localized to tumor tissues at a higher level than wild-type hPK5 (approximately 3-fold). Increased accumulation of NGR-hPK5 was correlated with stronger antiangiogenic effects *in vivo*, and only one-fifth the dose of NGR-hPK5 was needed for a similar antitumor effect produced by wild-type hPK5. These studies suggested that the antiangiogenic activity of hPK5 could be further improved by addition of an NGR motif.

## Materials and Methods

### Cell Culture

Mouse Lewis lung carcinoma (LLC) cells, human colorectal adenocarcinoma (Colo 205) cells and human umbilical vein endothelial cells (HUVECs) were purchased from the American Type Culture Collection (ATCC, Philadelphia, PA, USA). LLC and Colo 205 cells were grown in Dulbecco's modified Eagle's medium (DMEM) (HyClone, Logan, UT, USA) supplemented with 10% (v/v) fetal bovine serum (HyClone, Logan, UT, USA) and 1% penicillin-streptomycin (Invitrogen, Carlsbad, CA, USA). HUVECs were grown in Medium 200 (Cascade Biologics, Portland, OR, USA) supplemented with Low Serum Growth Supplement (LSGS). All cells were cultured in a humidified CO_2_ incubator at 37°C.

### Cloning of NGR-hPK5 Yeast Expression Plasmid

The plasmid pPIC9K-hPK5 for the expression of human plaminogen kringle 5 was constructed previously in our laboratory [28]. The Pichia pastoris yeast expression system was purchased from Invitrogen (Carlsbad, CA, USA). Restriction enzymes and Taq DNA polymerase were purchased from TaKaRa (Dalian, China). This clone was further modified to incorporate the CNGRC sequence at the amino terminus. The following sets of primers were used to modify hPK5 by PCR. NGR-hPK5 upper primer: 5′ CG CTCGAG AAA AGA TGC AAT GGT CGT TGC GGT GGT GGT GGT GTC CTG CTT CCA GAT GTA G 3′; lower primer: 5′ GC GAATTC TAG GCC GCA CAC TGA GGG AC 3′. Amplified fragments were purified by a DNA extraction kit, digested with Xho I and EcoR I, and then cloned into pPIC9K vector. Plasmid DNA was linearized at the Sac I site and used for homologous recombination into the yeast host strain GS115 (Invitrogen, Carlsbad, CA, USA) by electroporation.

### Expression and Purification of Recombinant NGR-hPK5 in Pichia Pastoris

Pichia clones were cultured in baffled shaker flasks and induced by methanol as described previously [28]. Methanol was supplemented daily to a final concentration of 0.5% during the post-culture period and cultured at 30°C for another 2 days with vigorous shaking. The clarified supernatant was collected and concentrated using ammonium sulfate precipitation (70% saturation), then dissolved in buffer A (20 mM Tris-HCl, 1 mM EDTA, 0.5 mM PMSF, pH 8.0), and finally dialyzed against the same buffer at 4°C. Proteins were purified by DEAE-Sepharose Fast Flow column (Pharmacia, Piscataway NJ, USA). After loading the sample, the column was washed with buffer B (20 mM Tris-HCl, 1 mM EDTA, pH 8.0) and eluted stepwisely with 0.1 M NaCl, 0.5 M NaCl in buffer B. The eluted protein fraction was analyzed by Tricine-SDS-PAGE (5% stacking gel and 16.5% separating gel). Protein concentration was determined by the Bradford assay (BioRad, Hercules, CA, USA).

### Cell Proliferation Assay

The effects of hPK5 and NGR-hPK5 on endothelial cell proliferation were assessed by the MTT assay. HUVECs in the exponential growth phase were seeded into a 96-well plate at a density of 5000 cells per well. After 24 h, hPK5 or NGR–hPK5 was added to a final concentration of 1, 5, 10 or 25 µg/ml respectively. The cells were incubated at 37°C for 48 h, then the cell viability was determined by the colorimetric MTT [3-(4, 5-dimethylthiazol-2-yl)-2, 5-diphenyl-2H-tetrazolium bromide] assay at wavelength 570 nm by TECAN Safire Fluorescence Absorbance and Luminescence Reader (Vienna, VA, USA). The cell viability was calculated according to the formula: Cell viability (%) = average A_570 nm_ of treated group/average A_570 nm_ of control group×100%.

### Cell Migration Assay

The effects of hPK5 and NGR-hPK5 on endothelial cell migration were assessed by the transwell assay and the wound healing assay. The cell migration assay was performed using transwell inserts (8.0 mm pore size, Millipore, Billerica, MA, USA) as described previously [30]. Before the experiment, HUVECs had been cultured in serum-free medium with hPK5 or NGR-hPK5 (PBS used as buffer control) at a concentration of 5 µg/ml for 16 h. Then the cells were harvested and re-suspended in the same medium. 1×10^5^ cells in a volume of 0.1 ml were added to the upper chamber, and the lower chamber was filled with 0.6 ml of 20% FBS supplemented medium. After incubation at 37°C for 9 h, cells on the upper surface of the membrane were removed. The migrant cells attached to the lower surface were fixed in 10% formalin at room temperature for 30 min, and stained for 20 min with a solution containing 1% crystal violet and 2% ethanol in 100 mM borate buffer (pH 9.0). The number of cells migrating to the lower surface of the membrane was counted in five fields under a microscope with a magnification of ×100. The wound healing assay was also performed as described previously [30]. Briefly, HUVECs plated onto fibronectin-coated (10 mg/ml) 24-well plates were serum-starved overnight, then wounded with a 200 ml pipette tip, washed with PBS, and incubated in the medium containing 10% FBS with hPK5 or NGR-hPK5 (PBS used as buffer control) at a concentration of 5 µg/ml for 20 h. The migration of the wounded cells was visualized and quantified under a microscope with a magnification of ×100. All groups of experiments were conducted in triplicate, and the cell number was counted by Image-Pro Plus 6.0 software.

### Cord Morphogenesis Assay

Matrigel (BD Biosciences, Bedford, MA, USA) was thawed at 4°C overnight and placed in a 96-well culture plate at 37°C for 1 h to allow gel formation. Before the experiment, HUVECs had been cultured in the medium with hPK5 or NGR-hPK5 (PBS used as buffer control) at a concentration of 10 µg/ml for 16 h. Then the cells were harvested, re-suspended in the same medium and seeded (45000 cells/cm^2^) on top of the solidified Matrigel. After incubation for 8 h at 37°C, the networks of cords were photographed in five fields under a microscope with a magnification of ×100. The total length of the cord structures in each photograph was measured by AxioVision 3.1 software (Carl Zeiss, Oberkochen, Germany). All groups of experiments were conducted in triplicate.

### Chick Embryo Chorioallantoic Membrane (CAM) Assay

The CAM assay was performed as described with slight modifications [31], [32]. Fertilized White Leghorn chicken eggs were placed in an incubator as soon as embryogenesis started and were kept under constant humidity at 37°C. Briefly, on day 8 the eggs were candled using a hand-held egg candler at the blunt end of the egg to identify the air sac and prominent blood vessels. Using a Dremel model drill (Dremel Racine, WI, USA), the CAM was separated from the shell by making a shallow burr hole at the blunt end of the egg. A solution of cortisone acetate (100 µg/disk, Sigma-Aldrich, St. Louis, MO, USA) was added to all disks in order to prevent an inflammatory response. Next, different concentrations of hPK5, NGR-hPK5 or buffer control were pipetted onto filter disks respectively, and the disk was then placed on the CAM in an avascular area. The window was sealed with sterile Scotch tape and the egg was returned to the incubator. After additional 2-day incubation, the possible antiangiogenic response was evaluated. CAM tissue directly below the filter disk was fixed with the mixture of methanol and acetone (1∶1) for 15 min. Tissues were washed 3 times with PBS and images were acquired using a stereomicroscope with photo-digital attachment. The response was scored as positive when CAM treated with the sample showed an avascular zone (≥5 mm in diameter) with very few vessels compared with the control group, and was calculated as the percentage of positive eggs relative to the total number of the eggs tested. Ten eggs were used for each group, and the data was reported as Mean ± SD based on results from three independent experiments.

### Ethics Statements

Six-week-old female C57BL/6J and athymic nude mice, which were purchased from the Vitalriver Animal Center (Vitalriver, Beijing, China), were housed in environmentally controlled conditions (22°C, a 12-h light/dark cycle with the light cycle from 6:00 to 18:00 and the dark cycle from 18:00 to 6:00) and maintained on standard laboratory chow. Animal welfare and treatment were carried out in strict accordance with the Guide for the Care and Use of Laboratory Animals (The Ministry of Science and Technology of China, 2006) and all experimental protocols were approved under animal protocol number SYXK(Su)2009-0017 by the Animal Care and Use Committee of College of Life Sciences, Nanjing University.

### *In Vivo* Animal Tumor Model Experiment

Female C57BL/6J and athymic nude mice (age 6 weeks) were obtained from the Vitalriver Animal Center and were acclimatized to local conditions for 1 week. Logarithmically growing mouse LLC and human Colo 205 cells were harvested by trypsinization and suspended in PBS at a density of 1×10^7^ cells/ml. Then, 100 µl of the single-cell suspension were injected subcutaneously into the right dorsum of C57BL/6J and nude mice. All tumor-bearing mice were divided randomly into groups of 8–10, and treatment was initiated on day 10 when the volume of tumor reached about 40–50 mm^3^. The mice were injected intraperitoneally (i.p.) with hPK5 or NGR-hPK5 daily. Tumor measurements were converted to tumor volume (V) as follows: L×W^2^×0.52, where L and W are the length and width, respectively. Measurements were taken by the Vernier caliper. All procedures followed approval of the Institutional Animal Care Committee. In a separate experiment cisplatin treatment was carried out in a regimen as described in results. Tumor sizes were shown as Mean ± SE and compared among groups using one-way analysis of variance (ANOVA). To determine whether hPK5/NGR-hPK5 in combination with cisplatin worked synergistically, the combination index (CI) was calculated as follows: CI = AB/(A×B). According to the tumor volume of each group, AB is the ratio of the combination group to the control group; A or B is the ratio of the single agent group to the control group. Thus a CI value less than, equal to or greater than 1 indicates that the drugs are synergistic, additive or antagonistic, respectively. A CI less than 0.7 indicates that the drugs are significantly synergistic.

### Tumor Localization

The uptakes in tumor of hPK5 and NGR-hPK5 were detected and compared by planar imaging and biodistribution studies. 1×10^6^ LLC cells were injected subcutaneously in the right front flank of female C57BL/6J mice (age 7 weeks). The mice were subjected to planar imaging and biodistribution studies when the tumor volume had reached 300–400 mm^3^ (2–3 weeks after inoculation).

#### Technetium-99 m labeling

Na^99 m^TcO_4_ solution (2.0 ml, >10 mCi/ml) was added to a lyophilized vial containing 0.455924 mg of NaH_2_PO_4_, 2.299752 mg of Na_2_HPO_4_, 40 µg of SnCl_2_, 10 µl of vitamin C, and 10 µg of hPK5 (or NGR-hPK5). The vial was placed into the lead pig and was allowed to stand at room temperature for 30 min. A sample of the resulting solution was analyzed by radio-HPLC. The radiochemical purity (RCP) was >95% for both ^99 m^Tc-hPK5 and ^99 m^Tc-NGR-hPK5 with a very small amount (<0.5%) of [^99 m^Tc] colloid.

#### Planar imaging

Ten tumor-bearing mice were randomly divided into two groups. Each mouse was administered with 500 µCi of ^99 m^Tc-hPK5 or ^99 m^Tc-NGR-hPK5 in 0.1 ml saline via tail vein and then anesthetized with isoflurane. The mice were placed prone on the pinhole collimator gamma camera (SIEMENS, symbia T6, Germany). Static images were acquired at 0.5, 1, 2, 3, 4 and 6 hours post-injection. The data were stored digitally in a 256×256 matrix, and the acquisition count limits were set at 300 sec. For data analysis, ROIs (regions of interest) were drawn over the tumor and the contralateral normal tissue. The SUVs (standardized uptake value) were acquired automatically by measuring the radioactivity in the region of interest and corrected for body weight and injected dose. The tumor-to-contralateral normal tissue (T/NT) ratios were calculated from the ROI analysis as Mean ± SD based on results from five tumor-bearing mice for each group.

#### Biodistribution studies

Thirty-six tumor-bearing mice were randomly divided into two groups. Each mouse was administered with 500 µCi of ^99 m^Tc-hPK5 or ^99 m^Tc-NGR-hPK5 in 0.1 ml saline via tail vein. Three mice of each group were sacrificed per time point at 0.5, 1, 2, 3, 4 and 6 hours post-injection, respectively. Blood samples were withdrawn with a syringe from the heart. The tumor and normal organs (heart, liver, spleen, lung, kidney, stomach, intestine, pancreas, brain, bone and muscle) were excised, washed with saline and weighed. The radioactivity was measured on a 1480 Wizard gamma counter (Perkin-Elmer). The organ uptake was calculated as the percentage of injected dose per gram of organ tissue (%ID/g). All radioactivity measurements were corrected for decay. The biodistribution data and T/NT (tumor-to-normal tissue) ratios were reported as Mean ± SD based on results from three tumor-bearing mice at each time point.

### Determination of Vessel Density

To determine the effect of the treatments on vessel density, immunofluorescence analysis was performed to visualize CD31-positive endothelial cells. LLC tumors for light microscopy were surgically resected and snap frozen. Frozen tumor sections (5 µm in thickness) were prepared according to standard protocols. Tumor sections (3 sections per mouse, totally 3 mice per group) were fixed in cold acetone (4°C) for 20 min, air dried, and blocked with 10% goat serum containing 1% BSA in PBS at room temperature for 30 min. Then the rat anti-mouse CD31 monoclonal antibody (BD Pharmingen, Franklin Lakes, NJ, USA) with a 1∶100 dilution was applied and incubated at 4°C overnight. Sections were rinsed in PBS 3 times for 5 min each. DyLight 594 goat anti-rat IgG (Jackson ImmunoResearch, West Grove, PA) secondary antibody with a 1∶500 dilution was applied at 37°C for 45 min in the dark. 4′, 6-Diamidino-2-phenylindole (DAPI, Santa Cruz Biotechnology, USA) counterstain was used to visualize nuclear detail. Images were acquired and processed by AxioVision 3.1 software on Carl Zeiss Axioplan 2. The number of microvessels in the field with the highest vessel density (‘hot spots’) was quantified according to the method as previously described [33]. Three fields with the highest vessel density per section were counted (with a magnification of ×200). Microvessel density was determined by averaging the number of microvessels in the counted fields.

### Data Analysis and Statistics

Values were presented as Mean ± SD or ± SE. For paired data, statistical analyses were performed using two-tailed Student's *t*-tests. For multiple comparisons, statistical analyses were performed using one-way analysis of variance (ANOVA) with a Tukey post-test. For all analyses, *p*<0.05 was considered statistically significant.

## Results

### Expression and Purification of NGR-hPK5

**Figure 1:**
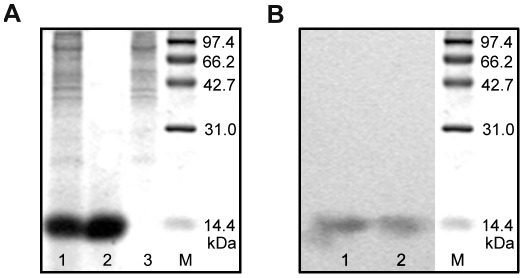
Recombinant NGR-hPK5 protein purification and identification. ***A*** **,** Expressed and purified NGR-hPK5 samples were separated by Tricine-SDS-PAGE, M: molecular weight markers. The culture supernatant was collected and concentrated using ammonium sulfate precipitation (70%), dissolved in buffer A (lane 1). After the ion exchange purification by DEAE-Sepharose Fast Flow column, NGR-hPK5 was eluted with 0.1 M NaCl in buffer B (lane 2), whereas the bulk impurities were eluted with 0.5 M NaCl in buffer B (lane 3). ***B*** **,** Western blot analysis of hPK5 (lane 1) and NGR-hPK5 (lane 2) proteins using anti-human plasminogen antibody.

The expression plasmid pPIC9K-NGR-hPK5, containing the cDNA encoding NGR-hPK5, was transfected into Pichia pastoris yeast strain GS115. The protein was purified by DEAE-Sepharose Fast Flow column. Purified protein was then analyzed by Tricine-SDS-PAGE and stained with Coomassie Blue (Fig. 1 A). The elution fraction was further examined by western blot using anti-human plasminogen antibody. As shown in Fig. 1 B, NGR-hPK5 migrated at 15 kDa as expected and no degradation was observed.

### Characterization of the Biological Activity of NGR-hPK5

**Figure 2:**
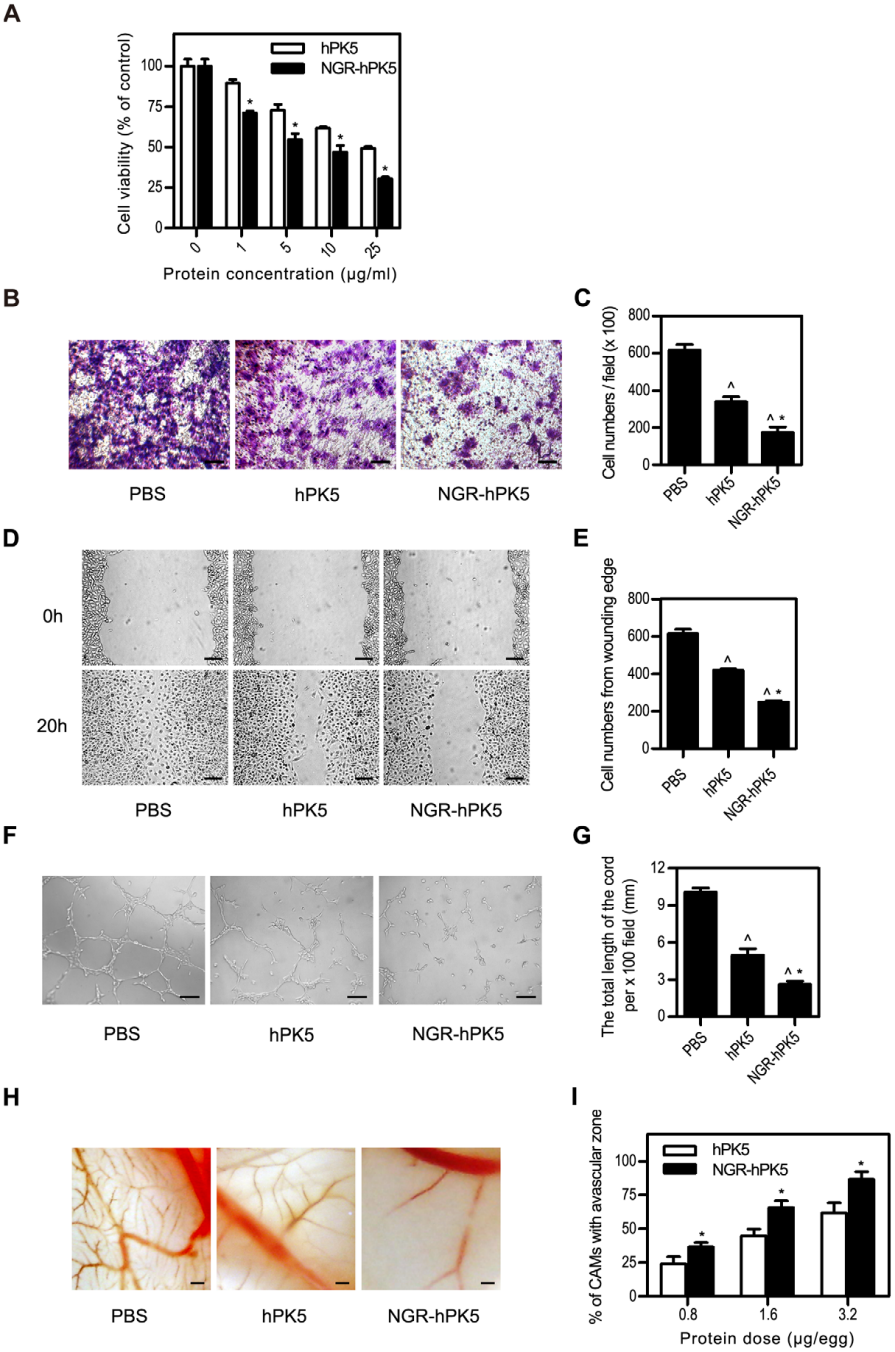
Antiangiogenic activity assessment of NGR-hPK5. ***A*** **,** MTT assay. HUVECs were treated with different concentrations of hPK5 or NGR-hPK5 (1, 5, 10 or 25 µg/ml) for 48 h. Cell viability (%) = average A_570 nm_ of treated group/average A_570 nm_ of control group×100%. N = 4. ***B*** & ***C*** **,** Transwell assay. HUVECs were treated with 5 µg/ml of hPK5 or NGR-hPK5. After 16 h pretreatment and 9 h incubation in the upper chamber, the cells migrating to the lower membrane were stained and counted in five fields with a magnification of ×100. N = 3, bar = 50 µm. ***D*** & ***E*** **,** Wound healing assay. HUVECs were treated with 5 µg/ml of hPK5 or NGR-hPK5. After 20 h healing period, the cells migrating into the wound area were visualized and quantified with a magnification of ×100. N = 3, bar = 100 µm. ***F*** & ***G*** **,** Cord morphogenesis assay. HUVECs were treated with 10 µg/ml of hPK5 or NGR-hPK5. After 16 h pretreatment and 8 h incubation on Matrigel, the networks of cords were photographed and the total length of the cord was measured in five fields under a microscope with a magnification of ×100. N = 3, bar = 100 µm. ***H*** & ***I*** **,** Chick CAM assay of angiogenesis. Representative CAMs from 8-day-old chick embryos, which were treated with different doses of hPK5 or NGR-hPK5 (0.8, 1.6 or 3.2 µg/egg) for 48 h. The data was calculated as the percentage of the positive eggs (formation of avascular zones ≥5 mm in diameter) relative to the total eggs tested. N = 10, bar = 600 µm. PBS was used as buffer control. All experiments were conducted in triplicate. * *p*<0.05 compared with hPK5, ∧ *p*<0.05 compared with control.

In order to assess the effect of NGR-hPK5 on angiogenesis *in vitro*, endothelial cell proliferation, migration and cord morphogenesis assays were performed. As shown in the MTT assay (Fig. 2 A), both hPK5 and NGR-hPK5 displayed a dose-dependent inhibitory effect on HUVEC proliferation, and NGR-hPK5 showed a more potent inhibitory effect than hPK5 (*p*<0.05). The concentration of hPK5 was about 25 µg/ml when inhibiting 50% HUVEC proliferation, while for NGR-hPK5 the ED_50_ was approximately 10 µg/ml. In addition to the anti-proliferation effect, NGR-hPK5 also showed more inhibitory effect on endothelial cell migration. Serum stimulated haptotaxis motility, measured by the transwell motility chamber assay, was used to examine the effect of NGR-hPK5 on HUVEC migration. The cells migrating to the lower membrane were stained and quantified as shown in Fig. 2 B&C. At the same dose, NGR-hPK5 showed more significant inhibition of cell migration than wild-type hPK5 (*p*<0.05, 5 µg/ml dose), which reduced the migration of HUVECs by 71.75% and 48.68% compared with control PBS group and hPK5 group, respectively. Meanwhile, the wound-healing scratch motility assay (Fig. 2 D&E) also revealed that, after 20 h healing period, NGR-hPK5 greatly reduced the migration of HUVECs as compared with hPK5 treated group or control PBS group at the same dose (5 µg/ml). The cells migrating into the wound area were counted. NGR-hPK5 treated group decreased the migrating cells by 59.34% compared with the control group, and 40.32% compared with hPK5 (*p*<0.05). Next, the effect of NGR-hPK5 on cord formation of endothelial cell was examined. HUVECs incubated on Matrigel for 8 h formed an extensive and enclosed capillarylike structure. hPK5 and NGR-hPK5 (both at 10 µg/ml dose) impaired the ability of HUVECs to form this structure, resulting in an incomplete and sparse cord network (Fig. 2 F&G). NGR-hPK5 treated group showed more significant inhibition than hPK5 group, the cord formation in NGR-hPK5 group was decreased by 73.88% compared with control PBS group, and 47.25% compared with hPK5 group (*p*<0.05). Subsequently, chick embryo CAM model was used to evaluate the antiangiogenic activity of NGR-hPK5 *in vivo*. Dried filter disks, adsorbed with hPK5 or NGR-hPK5 at 0.8, 1.6 and 3.2 µg/embryo doses, were implanted on the top of growing CAMs. Two days later, hPK5 and NGR-hPK5 induced a strong antiangiogenic response in the CAM tissues in a dose dependent manner (Fig. 2 H&I), as shown by the decreased number of branching vessels in the center of the filter disk. At the same dose, NGR-hPK5 showed increased antiangiogenic activity compared with the wild-type hPK5 (*p*<0.05). In sum, these data suggested that an additional NGR modification to hPK5 could improve its biological activity of antiangiogenesis *in vitro* and *in vivo*.

### Inhibition of Tumor Growth

**Figure 3:**
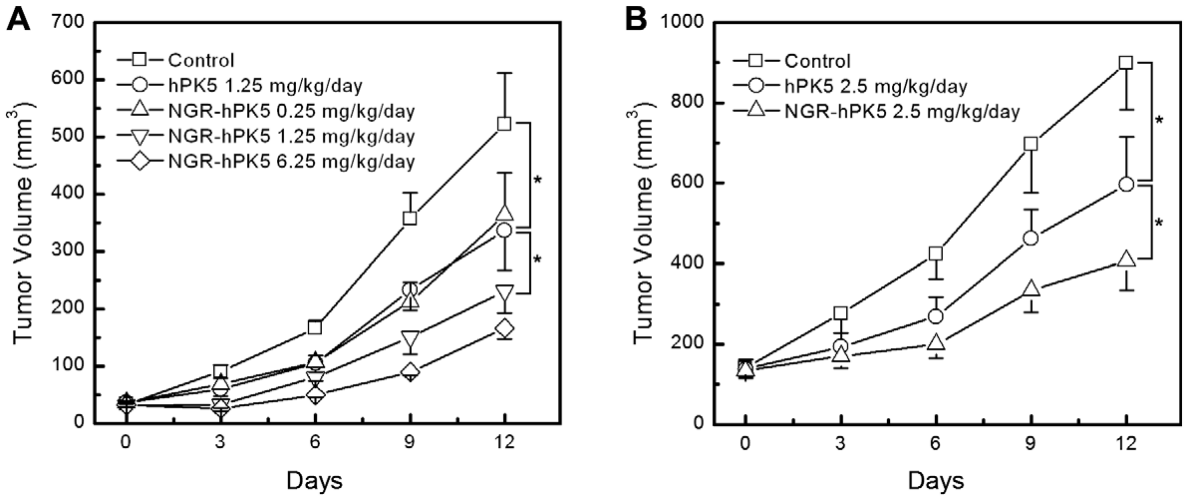
Improved inhibition of LLC and Colo 205 Tumors by NGR sequence modified hPK5 (NGR-hPK5). To determine whether NGR-hPK5 could improve the antitumor activity of hPK5, two tumor model systems were employed. ***A*** **,** The first tumor model system used syngeneic C57BL/6J mice bearing LLC tumors. From day 0 on, the mice were injected i.p. daily for 5 days with NGR-hPK5 at 0.25, 1.25 and 2.5 mg/kg/day dose, or with hPK5 at 1.25 mg/kg/day dose. Both hPK5 and NGR-hPK5 significantly inhibited the growth of LLC solid tumors. At the same dose of 1.25 mg/kg/day, NGR-hPK5 resulted in more significant inhibition of tumor growth than wild-type hPK5. ***B*** **,** The second tumor model system used athymic nude mice bearing Colo 205 xenografts. Administered i.p. daily for 5 days at 2.5 mg/kg/day dose, the *in vivo* antitumor activity of NGR-hPK5 was higher than that of hPK5 on day 12. Tumor volume was calculated by the formula (L×W^2^×0.52). Eight mice were used in each sample unit, and the data shown were the mean volume ± SE. * *p*<0.05.

To determine whether NGR-hPK5 could improve the antitumor activity of hPK5, we used two tumor model systems. The first model system used the mouse Lewis lung carcinoma (LLC) cell line (Fig. 3 A). From day 0 on, the mice were injected i.p. daily for 5 days with NGR-hPK5 at 0.25, 1.25 and 2.5 mg/kg/day dose, or with hPK5 at 1.25 mg/kg/day dose. Both hPK5 and NGR-hPK5 significantly inhibited the growth of LLC solid tumors (on day 3–12, *p*<0.05, compared with control). On day 12, the mean volume of tumor was 336.17±69.15 mm^3^, with 35.52% inhibition when using hPK5 at 1.25 mg/kg/day dose. Treatment with NGR-hPK5 resulted in dose-dependent inhibition of tumor growth, with 30.30%, 55.68% and 68.10% inhibition observed at 0.25, 1.25 and 2.5 mg/kg/day dose respectively. At the same dose NGR-hPK5 resulted in more significant inhibition of tumor growth than wild-type hPK5 (*p*<0.05, compared with wild-type hPK5 at 1.25 mg/kg/day dose), while the antitumor activity of NGR-hPK5 at 0.25 mg/kg/day dose was similar to that of hPK5 at 5-fold excess, i.e., hPK5-treated group at 1.25 mg/kg/day dose. The second model used the human colorectal adenocarcinoma cell line, Colo 205, in athymic nude mice (Fig. 3 B). NGR-hPK5 and hPK5 were administered i.p. daily for 5 days at 2.5 mg/kg/day dose. In this model system, i.p. injection of wild-type hPK5 inhibited tumor growth by 33.75% on day 12 (595.76±119.10 mm^3^). Under similar conditions, NGR-hPK5 treatment showed 54.76% inhibition (on day 9 and 12, *p*<0.05, compared with wild-type hPK5).

### Increased Tumor Localization of NGR-hPK5

**Figure 4:**
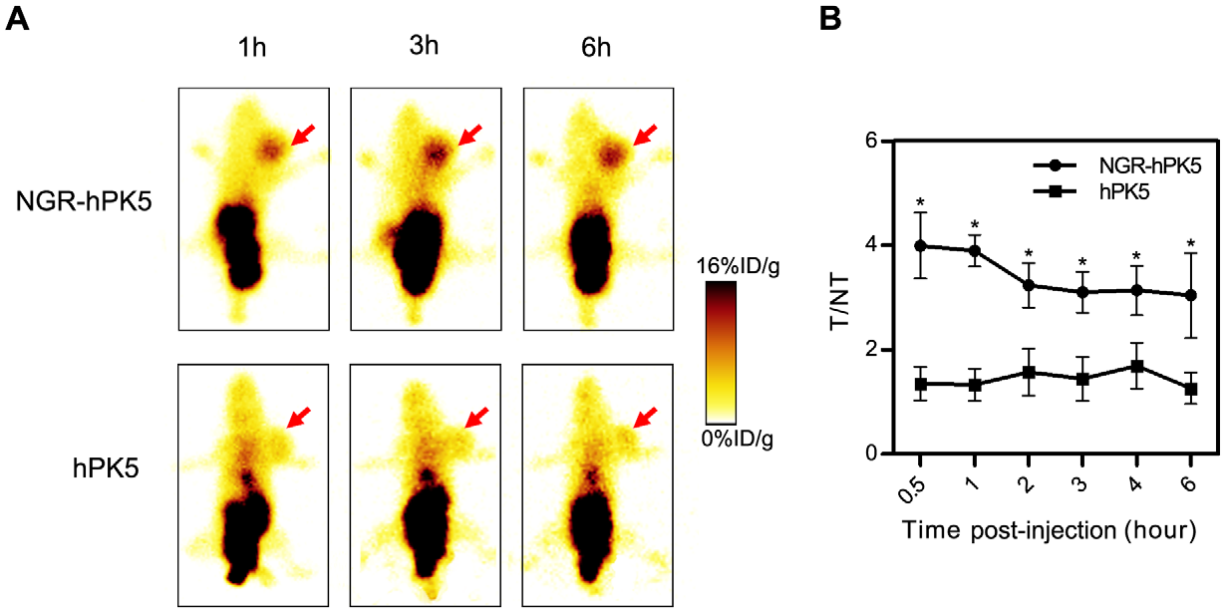
Detection of hPK5 and NGR-hPK5 in tumor localization by planar imaging analysis. When the LLC tumor volume reached about 300–400 mm^3^, ^99 m^Tc-labeled hPK5 or NGR-hPK5 (500 µCi) were injected i.v. into LLC grafting C57BL/6J mice. Planar images were acquired in the prone position at 0.5, 1, 2, 3, 4 and 6 hours post-injection. ***A*** **,** Representative images from the planar imaging of ^99 m^Tc-NGR-hPK5 and ^99 m^Tc-hPK5 at 1, 3 and 6 hours post-injection. (arrows LLC tumors) ***B*** **,** The tumor-to-contralateral normal tissue (T/NT) ratios were calculated from the ROI analysis. N = 5. * *p*<0.05 compared with hPK5.

**Figure 5:**
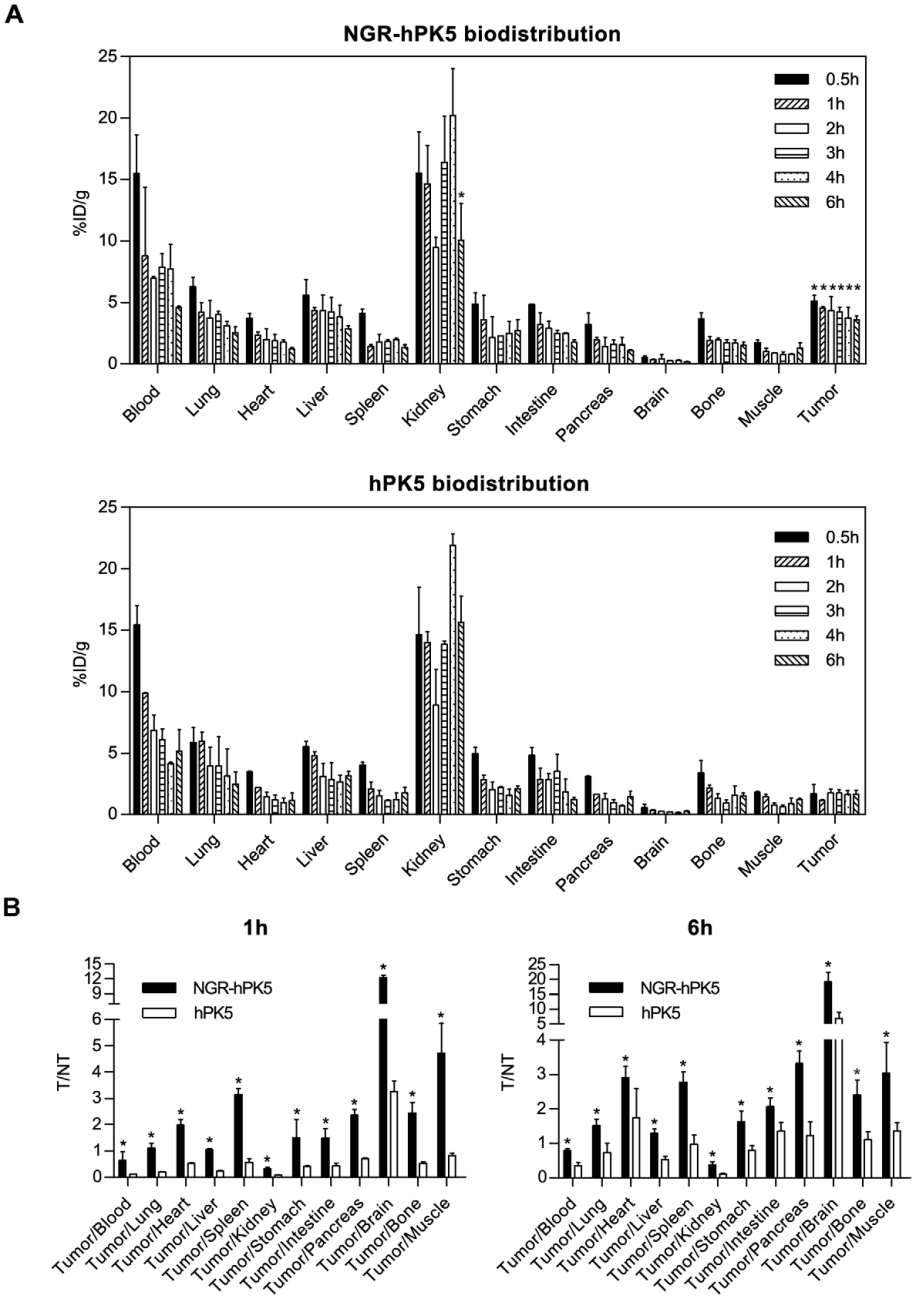
Biodistribution analysis of hPK5 and NGR-hPK5 in tumor-bearing mice. When the LLC tumor volume reached about 300–400 mm^3^, ^99 m^Tc-labeled hPK5 or NGR-hPK5 (500 µCi) were injected i.v. into LLC grafting C57BL/6J mice. Tumor, blood and major organs were collected, weighed and counted on a gamma counter at 0.5, 1, 2, 3, 4 and 6 hours post-injection. ***A*** **,** The biodistribution of ^99 m^Tc-labeld NGR-hPK5 and hPK5 in tumor, blood and normal organ (lung, heart, liver, spleen, kidney, stomach, intestine, pancreas, brain, bone and muscle) at 0.5, 1, 2, 3, 4 and 6 hours post-injection. The organ uptake was calculated as the percentage of injected dose per gram of organ tissue (%ID/g). The tumor uptake of NGR-hPK5 was significantly higher (p<0.05) than that of hPK5. No statistically significant difference has been found in their respective distribution in other tissues except for kidney in which the level of NGR-hPK5 was lower than that of hPK5 at 6 h. N = 3. ***B*** **,** The tumor-to-normal tissue ratios of NGR-hPK5 and hPK5 at 1 h and 6 h post-injection were calculated from ***A***. * *p*<0.05 compared with hPK5.

To assess whether the improved endothelial cell binding *in vitro* could translate into enhanced tumor homing *in vivo*, tumor localization studies were performed. ^99 m^Tc-labeled hPK5 or NGR-hPK5 was injected i.v. into LLC grafting C57BL/6J mice. Planar images were acquired at 0.5, 1, 2, 3, 4 and 6 hours post-injection. As shown in Fig. 4 A, NGR-hPK5 exhibited an obviously higher accumulation in tumors than hPK5 did at each time point. The uptake in the tumor and its contralateral normal tissue was measured from the ROI analysis and shown in Fig. 4 B, the tumor-to-normal tissue (T/NT) ratios of NGR-hPK5 was 3.0–4.0, while the ratios of hPK5 was 1.3–1.7. Subsequently, the biodistribution studies of ^99 m^Tc- NGR-hPK5 and ^99 m^Tc-hPK5 were performed. Tumor, blood and major organs were collected, weighed and counted on a gamma counter at the same time schedule as described in planar imaging. The data was presented as the percentage injected dose per gram of tissue (%ID/g) in Fig. 5A, the tumor uptake of NGR-hPK5 was from 5.11±0.46%ID/g to 3.58±0.31%ID/g during 0.5 h to 6 h post-injection, while the tumor uptake of hPK5 was from 1.77±0.28%ID/g to 1.15±0.06%ID/g. Consistent with the results of planar imaging, the *in vivo* NGR-hPK5 tumor uptake was significantly higher than that of hPK5 (approximately 3-fold). Both of the proteins were excreted mainly through the kidneys, the levels of NGR-hPK5 and hPK5 in kidneys were similar from 0.5 h to 4 h post-injection, but the level of NGR-hPK5 (10.06±2.98%ID/g) was lower than that of hPK5 (15.65±2.13%ID/g) by the end of 6 h. No statistically significant difference has been found for their respective distribution in blood, lung, heart, liver, spleen, stomach, intestine, pancreas, brain, bone or muscle. The tumor-to-normal tissue ratios of NGR-hPK5 and hPK5 from the data of biodistribution studies were calculated and compared in Fig. 5 B. The tumor/blood, tumor/lung, tumor/heart, tumor/liver, tumor/spleen, tumor/kidney, tumor/stomach, tumor/intestine, tumor/pancreas, tumor/brain, tumor/bone and tumor/muscle ratios of NGR-hPK5 were all significantly higher (*p*<0.05) than those of hPK5 at 1 h and 6 h. Taken together, NGR-hPK5 showed selective targeting in the LLC tumor and did not lead to nonspecific accumulation in other tissues.

### Suppression of Tumor Neovascularization

**Figure 6:**
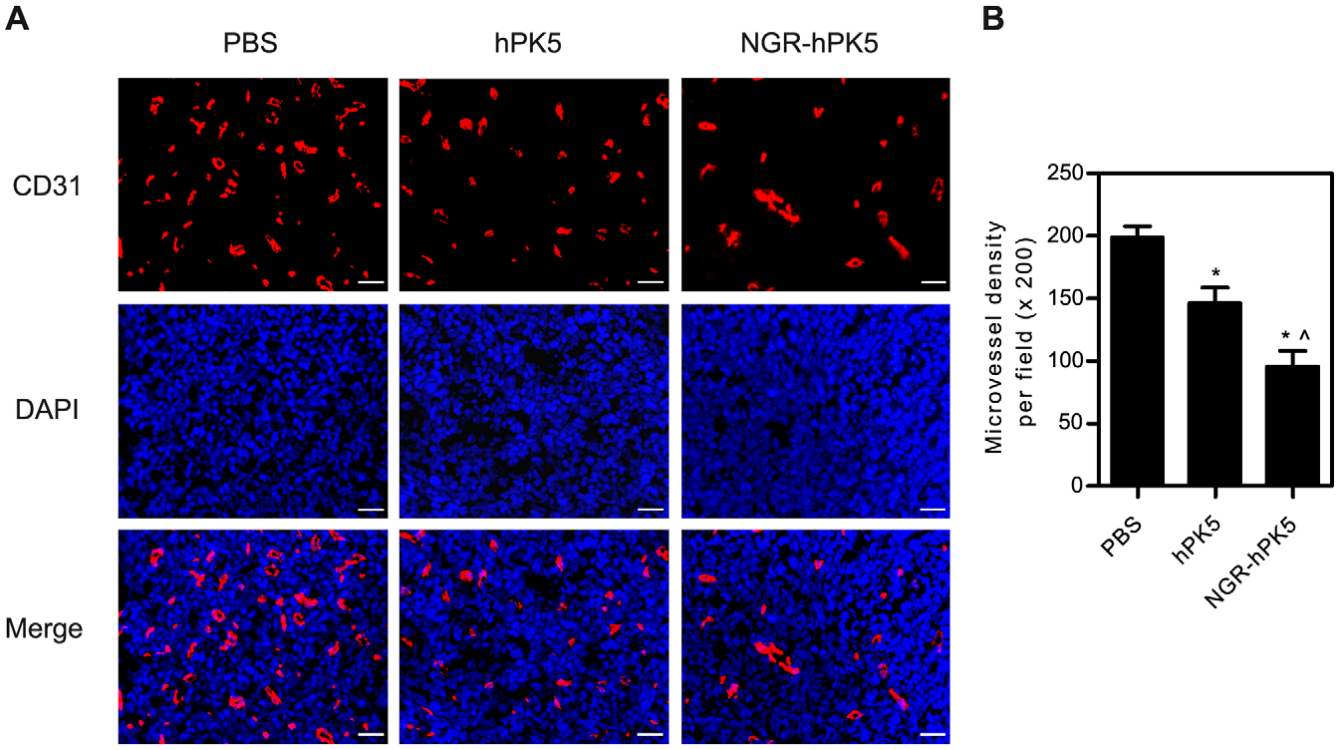
Immunofluorescence analysis of neovascularization in tumors. LLC tumor-bearing C57BL6/J mice were systemically treated with control saline or with 2.5 mg/kg/day of hPK5 or NGR-hPK5 daily for 5 days, and primary tumors were resected on day 1 after post-treatment. ***A*** **,** The immunofluorescence analysis was evaluated for visualization of CD31-positive endothelial cells. DAPI counterstain was used to visualize nuclear detail. ***B*** **,** Microvessel density per field (×200) of experimental group and saline-treated group. The data shown were the mean ± SD. Microvessels were counted from three most vascular areas (‘hot spots’) fields in tumors from three mice in each group. Bar = 20 µm, * *p*<0.05 compared with control, ∧ *p*<0.05 compared with hPK5.

To determine the effects of treatment on early tumor neovascularization, we examined blood vessel density in tissue sections from LLC tumor using anti-mouse CD31 antibody and standard immunofluorescence techniques. LLC tumor-bearing C57BL6/J mice were systemically treated with control saline or with 2.5 mg/kg/day of hPK5 or NGR-hPK5 daily for 5 days, and primary tumors were resected on day 1 after post-treatment. The microvessel density (MVD) was estimated by the mean of CD31-positive endothelial cells from three most vascular areas (‘hot spots’) within the tumor section. Fig. 6 showed that wild-type and NGR modified hPK5 treatment resulted in reduced microvessel density by 26.34% and 51.85% compared with control respectively (*p*<0.05). The NGR motif enhanced the antiangiogenic effect of hPK5 on the density of blood vessels significantly, which reduced microvessel density by 34.62% in comparison with wild-type hPK5 (*p*<0.05).

### Combination of Cisplatin and hPK5/NGR-hPK5 Inhibits Tumor Growth Synergistically

**Figure 7:**
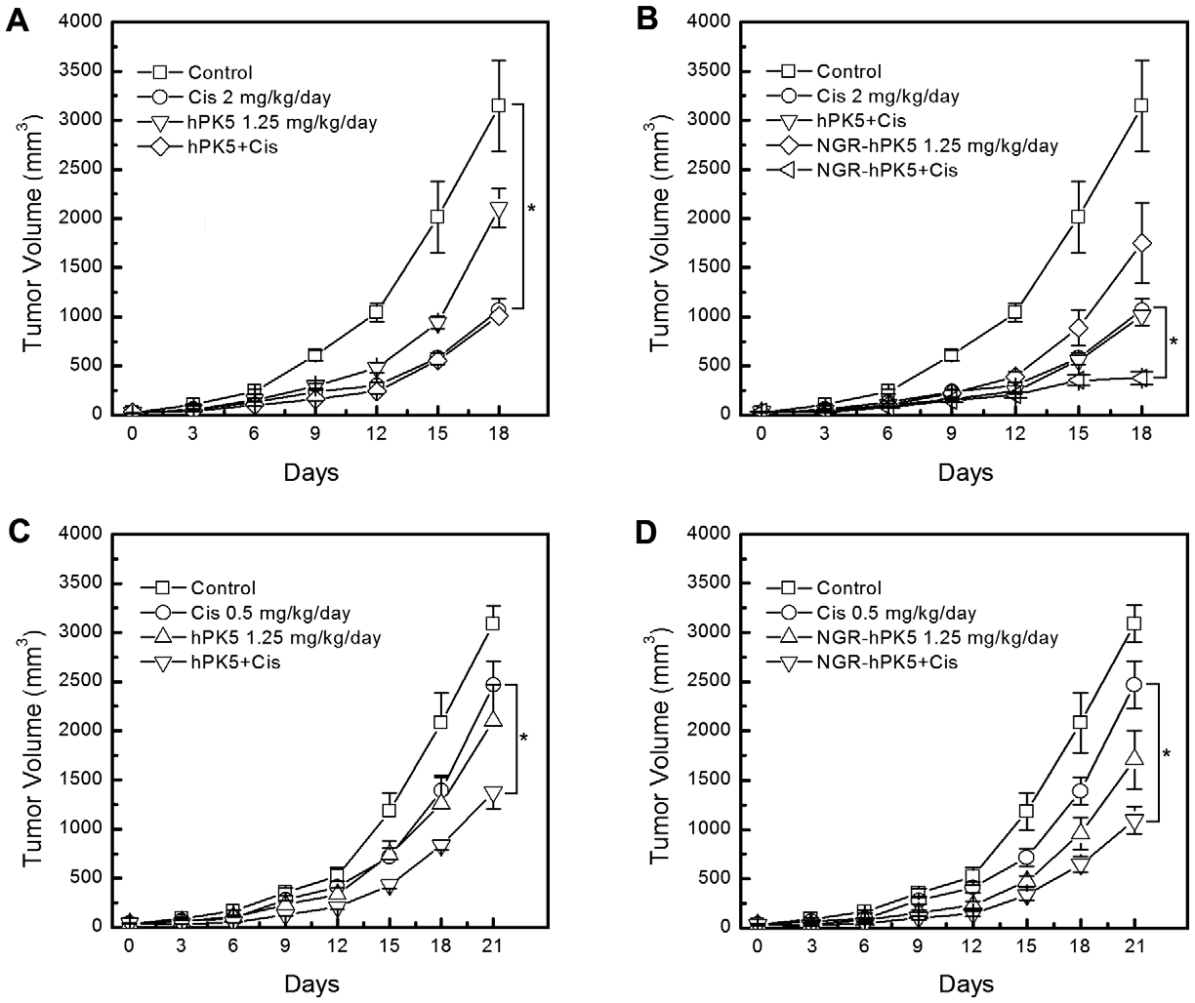
Tumor growth suppression in C57BL/6J mice bearing LLC tumors treated with hPK5/NGR-hPK5 and cisplatin. When the tumor size reached about 50 mm^3^, the mice were injected i.p. every other day with hPK5/NGR-hPK5 (on days 0, 2, 4, 6, 8 and 10; 6 times totally) and 24 h after each treatment mice were injected i.p. with cisplatin (on days 1, 3, 5, 7, 9 and 11; 6 times totally). The dose of hPK5 or NGR-hPK5 was 1.25 mg/kg/day in all protein therapy groups. Control mice were treated with injections of PBS. ***A*** **,** hPK5 combined with 2 mg/kg/day of cisplatin; ***B*** **,** NGR-hPK5 combined with 2 mg/kg/day of cisplatin; ***C*** **,** hPK5 combined with 0.5 mg/kg/day of cisplatin; ***D*** **,** NGR-hPK5 combined with 0.5 mg/kg/day of cisplatin. Tumor volume was calculated by the formula (L×W^2^×0.52). Eight to ten LLC tumor-bearing mice were used in each sample unit, and the data shown were the mean volume ± SE. * *p*<0.05.

To determine whether hPK5/NGR-hPK5 could improve the antitumor activity of cisplatin chemotherapy, we used the LLC tumor model system. From day 0 on, the mice were injected i.p. every two days with hPK5 or NGR-hPK5 at 1.25 mg/kg/day dose (on days 0, 2, 4, 6, 8 and 10). On the following day after each injection with the proteins, different doses of cisplatin were administered i.p. to the mice (on days 1, 3, 5, 7, 9 and 11). On day 18, a significant antitumor response was observed with 66.00% inhibition when mice received cisplatin alone at a high dose (2 mg/kg/day) (*p*<0.05, compared with control), while the effect of hPK5 plus cisplatin (Fig. 7 A) resulted in 67.83% inhibition of tumor growth compared with control, which had no statistically significant difference from that of cisplatin alone (*p*>0.05). However, as shown in Fig. 7 B, NGR-hPK5 could enhance the antitumor effect of cisplatin at the same dose. On day 18, the mean tumor size in combination therapy group was significantly reduced to 11.34% of the control group and 33.45% of the cisplatin-treated group (*p*<0.05, compared with cisplatin). The combination index (CI) was 0.636, which indicated that the drugs were significantly synergistic. Cisplatin using at a low dose (0.5 mg/kg/day) induced border or marginal effect, but the antitumor effect of recombinant protein plus cisplatin was stronger than that of protein alone or cisplatin alone. As shown in Fig. 7 C, on day 21, cisplatin or hPK5 alone therapy group resulted in 20.04% and 31.89% inhibition of tumor growth compared with the control group, while combination therapy of hPK5 and cisplatin resulted in 55.46% and 59.92% inhibition of tumor growth compared with the control group and cisplatin-treated group (*p*<0.05). NGR-hPK5 therapy alone showed 44.65% inhibition of tumor growth compared with control on day 21 (Fig. 7 D). NGR-hPK5 in combination with cisplatin produced strong antitumor response with 64.52% and 68.94% inhibition compared with the control group and cisplatin-treated group (*p*<0.05). The CI value of hPK5 or NGR-hPK5 combination with cisplatin (at a low dose of 0.5 mg/kg/day) was 0.818 and 0.802 respectively, which indicated that they all had a synergistic effect.

**Figure 8:**
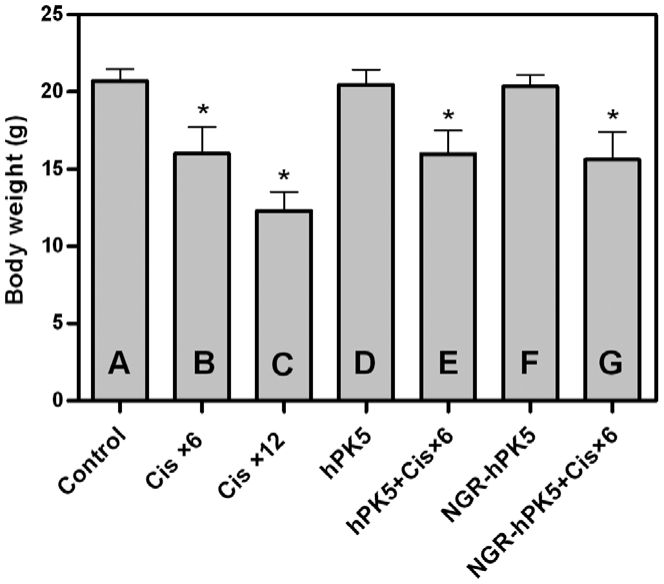
The effect on body weight analysis of hPK5/NGR-hPK5 and cisplatin. C57BL/6J mice were injected i.p. with either PBS or cisplatin, recombinant protein alone or combined with cisplatin. On day 1 after post-treatment, the body weight was observed. ***A*** **,** Control (PBS); ***B*** **,** Cisplatin at 2 mg/kg/day dose every other day (6 times totally); ***C*** **,** Cisplatin at 2 mg/kg/day dose every day (12 times totally); ***D*** **,** hPK5 at 1.25 mg/kg/day dose every other day (6 times totally); ***E*** **,** hPK5+cisplatin (B); ***F*** **,** NGR-hPK5 at 1.25 mg/kg/day dose every other day (6 times totally); ***G*** **,** NGR-hPK5+cisplatin (B). Five mice were used in each sample group, and the data shown were the mean volume ± SD. * *p*<0.05 compared with control.

To investigate whether the combination therapeutic activity occurred at the expense of additional toxicity, C57BL/6J mice were injected i.p. with either PBS or cisplatin, recombinant protein alone or a combination of both recombinant protein and cisplatin. As shown in Fig. 8, treatment with hPK5 or NGR-hPK5 was well tolerated at the dose tested, whereas treatment with cisplatin at a high dose (2 mg/kg/day) 6 times and 12 times caused 22.62% and 40.60% weight loss respectively compared with the control group. Cisplatin exhibited antitumor activity *in vivo*, but weight loss was also observed. Combination therapy of recombinant protein (hPK5 or NGR-hPK5) and cisplatin did not result in additional weight loss compared with cisplatin treatment. These results suggested that hPK5 or NGR-hPK5 could enhance antitumor effects of cisplatin without causing significant weight loss.

## Discussion

Angiogenesis plays a key role in tumor progression [34]. It was hypothesized that inhibition of angiogenesis would be an effective strategy to treat human cancer, and an active search for angiogenesis inducers and inhibitors began in 1971 [34]. Several endogenous angiogenesis inhibitors are protein fragments derived from extracellular matrix [35] or hemostatic system proteins [36]. Plasminogen is a blood protein that is proteolysed into potent angiogenesis inhibitors, such as angiostatin (kringles 1–4), kringles 1–5, kringles 1–3, and kringle 5 [12]. Among them, K5 displays the most potent inhibitory activity to endothelial cell proliferation and migration [15], [16] among known naturally occurring angiogenesis inhibitors. Several reports have shown that hPK5 has a potential therapeutic effect in angiogenesis-related diseases, including solid tumors. For instance, Perri et al observed that in a nude mouse orthotopic brain cancer model tumor-targeted hPK5 expression was capable of effectively suppressing glioma growth and promoting significant long-term survival (>120 days) of test animals [25]. The hPK5 induced a marked reduction in blood vessel formation and significantly suppressed the recruitment of tumor-infiltrating CD45^+^ Mac3^+^ Gr1^−^ macrophages [25]. Successive studies have suggested that hPK5 could have therapeutic potential in hepatocellular carcinoma [20]–[22], lung cancer [23], [24], glioblastoma [25], [26], ovarian cancer [27] etc. In our previous study, we explored the therapeutic alliance of radiotherapy and hPK5 to inhibit the LLC tumor growth in tumor-bearing animals [28]. The results indicated that there was a significant synergistic effect between radiotherapy and hPK5 antiangiogenesis treatment, compared with each single treatment method. The mechanism of synergy might be due to that hPK5 increased the sensibility of both LLC and vascular endothelial cells to ionizing radiation. It has been suggested that hPK5 acts as a novel anti-cancer agent, resulting in a potent, clinically relevant antitumor effect.

In the current study, coupling hPK5 with NGR peptide improved its antineoplastic activity and only low doses of NGR-hPK5 were needed for effective therapy by the vascular targeting strategy. Because the function of NGR peptide is dependent on its conformational characteristics, we simulated the three-dimension structure of hPK5 using Cn3D 4.1 program to determine the spatial positions of amino and carboxyl terminus. Both amino and carboxyl terminus were localized at the surface of the molecular structure (data not shown). Therefore NGR peptide being fused to NH_2_- or -COOH terminus of hPK5 could not have a great effect on its binding activity to CD13.

Giorgio et al [37] investigated the structure and tumor-homing properties of cyclic CNGRC-TNF α (containing disulfide bridge) and linear GNGRG-TNF α conjugates, and compared their antitumor activity. Experiments carried out in animal models showed that both linear GNGRG and cyclic CNGRC could target TNF α to tumors. However, the antitumor activity of CNGRC-TNF α was over 10 times higher than that of GNGRG-TNF α. The molecular dynamic simulation showed that the NGR motif had a strong propensity to form β-turn (Gly^3^-Arg^4^) in linear peptides, and the disulfide bridge constraint was critical for stabilizing the bent conformation and for increasing the tumor targeting efficiency. In the present study, cyclic CNGRC was selected to modify hPK5 at its amino terminus via a Gly_4_ linker to ensure that the function of NGR peptide and hPK5 could not influence each other.

APN expresses at a high level in tumor vasculature and plays an important role in angiogenesis [2]. APN is up-regulated in response to hypoxia and to angiogenic growth factors, such as basic fibroblast growth factor (bFGF) and vascular endothelial growth factor (VEGF), and its signals regulate capillary tube formation during angiogenesis [38]. Moreover, studies have revealed that APN/CD13 is a marker for semiquiescent cancer stem cells (CSCs), and its elevated expression correlates with tumor metastasis and unfavorable prognosis [39]–[41]. Antibodies and functional inhibitors to APN blocked retinal neovascularization, chorioallantoic membrane angiogenesis, and tumor growth [2]. The addition of an NGR-sequence at the amino terminus of endostatin resulted in strong binding and inhibition of endothelial cell APN [11]. Yokoyama et al reported that NGR-endostatin showed increased binding to endothelial cells and had higher tumor localization than the native protein, and increased binding of endostatin also coincided with improved antiangiogenic properties of endostatin [11].

Therefore, addition of a peptide that contains NGR could promote both NGR-dependent and -independent signaling via APN/CD13, resulting in potent antiangiogenic activity of hPK5. In this study, hPK5 was genetically modified to introduce an NGR motif (NGR-hPK5) and was expressed in GS115. The effect of NGR-hPK5 treatment on early tumor neovascularization was examined by measurement of microvessel density (Fig. 6). Hlatky et al [42] caution that although microvessel density is a useful prognostic marker, it is not, by itself, an indicator of therapeutic efficacy. Microvessel density alone is insufficient to distinguish between an angiogenic activity that is directly disrupting pathways governing vessel growth and an activity that alters the metabolic burden of the supported tumor cells. Thus additional assays for evaluating the antiangiogenic efficacy of NGR-hPK5 were included. The biological activity of NGR-hPK5 was assessed and compared with that of hPK5 by endothelial cell proliferation, migration, cord morphogenesis assays and CAM assay (Fig. 2). NGR-hPK5 exhibited directly increased antiangiogenic activity *in vitro* and *in vivo*. Our data (Fig. 4 and Fig. 5) also showed that NGR-hPK5 was localized to tumor tissues at a higher level than wild-type hPK5. ^99 m^Tc-labeled hPK5 and NGR-hPK5 were determined in tumor and major organs by planar imaging and biodistribution studies from 0.5 h to 6 h post-injection. The tumor uptake of NGR-hPK5 was significantly higher than that of hPK5 at each time point (approximately 3-fold). Increased accumulation of NGR-hPK5 correlated with stronger antiangiogenic effects *in vivo*. Only one-fifth the dose of NGR-hPK5 was needed for a similar antitumor effect produced by wild-type hPK5. These studies indicated that NGR modification could enhance antiangiogenesis activity of hPK5 by targeted delivery to the tumor vasculature and improved the antitumor activity of hPK5.

Preclinical studies have shown that *in vivo* frequently protracted administration of low dosages of conventional chemotherapeutic drugs on a metronomic or antiangiogenic schedule could also damage or kill the endothelial cells of tumor neovasculature and delay acquired resistance to these chemotherapeutic drugs [43]–[45]. Tan et al [44] explored the efficacy of a strategy combining low-dose cisplatin and a recombinant xenogeneic endoglin as an antiangiogenic protein vaccine. The combination therapy resulted in not only significant antiangiogenic effects but also additional promotion of tumor cell apoptosis and inhibition of tumor cell proliferation, without any ensuing increase in host toxicity during treatment. In addition, the combination demonstrated a synergistic relationship, which was shown in all of the synergistic indexes, i.e., tumor volume, angiogenesis, apoptosis and proliferation. Other findings have also suggested that vascular targeting could increase vascular permeability, alter tumor barriers and increase the penetration of chemotherapeutic drugs [46], [47]. Cisplatin is widely used in the treatment of human tumors [48]. However, the potential for tumor control with cisplatin chemotherapy must always be carefully balanced with the risk for normal tissue damage [49], [50]. In the current study, though cisplatin at a high dose could produce a significant antitumor response, significant systemic toxicity such as weight loss was observed. Cisplatin at a low dose exerted modest antitumor effect with decreased toxicity, whereas cisplatin in combination with hPK5 or NGR-hPK5 significantly enhanced the therapeutic effect. At the same dose, combination therapy with NGR-modified hPK5 and cisplatin resulted in a stronger inhibition of tumor growth than the combination therapy with hPK5 and cisplatin. It indicated that vascular targeting could be a novel strategy for increasing the therapeutic index of chemotherapeutic drugs.

In our studies, we successfully expressed NGR-hPK5 in yeast and purified the new protein. *In vitro* and *in vivo* NGR-hPK5 had stronger antiangiogenesis activity than wild-type hPK5, which indicated that NGR modification of antiangiogenic molecules, such as hPK5, could be used to improve their therapeutic efficacy.
